# Mitochondrial Coenzyme Q Protects Sepsis-Induced Acute Lung Injury by Activating PI3K/Akt/GSK-3*β*/mTOR Pathway in Rats

**DOI:** 10.1155/2019/5240898

**Published:** 2019-11-13

**Authors:** Ruirui Li, Tao Ren, Jianqiong Zeng

**Affiliations:** ^1^Department I of Critical Care Medicine, The First Affiliated Hospital of the Medical College, Shihezi University, North Second Road, Shihezi, Xinjiang Uygur Autonomous Region, 832008, China; ^2^Departments III of Cardiology, The First Affiliated Hospital of the Medical College, Shihezi University, North Second Road, Shihezi, Xinjiang Uygur Autonomous Region, 832008, China; ^3^Departments of Cardiovascular Surgery, First People's Hospital of Foshan, No. 81 Lingnan Avenue North, Chancheng District, Foshan, Guangdong Province, 528000, China

## Abstract

The aim of our study was to assess the effects of mitochondrial coenzyme Q (MitoQ) on sepsis-induced acute lung injury (ALI) and investigate its possible mechanisms. The cecal ligation and puncture (CLP) method was used to establish a septic ALI model. Rats were randomly divided into Con group, CLP group, MitoQ group, and MitoQ + LY294002 group. The survival rate of the rats was recorded, and the survival rate curve was plotted. Moreover, the ratio of wet/dry weight (W/D) in lung tissue was measured. The activity of myeloperoxidase (MPO) was measured by using the MPO colorimetric activity assay kit. The levels of high-mobility group box 1 (HMGB1) and interleukin-6 (IL-6), macrophage inflammatory protein 2 (MIP2), and keratinocyte chemoattractant (KC) were analyzed by ELISA. The histopathological changes were measured by HE staining, and the lung injury was scored. TUNEL assay was applied to detect the apoptotic cells in lung tissue. The protein expressions were detected by western blot. MitoQ increased the survival rate and alleviated pulmonary edema in septic ALI rats. In addition, MitoQ inhibited the MPO activity and decreased the levels of HMGB1 and IL-6. After treatment with MitoQ, alveolar wall edema, inflammatory cell infiltration, and red blood cell exudation were relieved. MitoQ inhibited cell apoptosis in lung tissue of septic ALI rats. Meanwhile, MitoQ treatment remarkedly increased the expression of p-Akt, p-GSK-3*β*, and p-mTOR but decreased Bax, caspase-3, caspase-9, Beclin-1, and LC-3II/LC-3I. The effects of MitoQ were significantly reversed by the PI3K inhibitor (LY294002). Our study demonstrated that MitoQ could protect sepsis-induced acute lung injury by activating the PI3K/Akt/GSK-3*β*/mTOR pathway in rats.

## 1. Introduction

Sepsis, a common acute and critical disease, is mainly characterized by sustained hypotension, metabolic acidosis, and systemic inflammatory response syndrome [1]. It can lead to multiple organ injuries and even death. Due to the pulmonary susceptibility, acute lung injury (ALI) is one of the common lethal complications of sepsis [2]. Although numerous studies focus on investigating the diagnosis and pathogenesis of sepsis-induced ALI, current treatment protocol is still limited. Thus, searching for novel and more effective therapeutic medicines will be helpful for the therapy of sepsis-induced ALI.

Mitochondrial coenzyme Q (MitoQ) is a lipid-soluble membrane component, which widely existed in the organisms ranging from bacteria to mammals [3]. Accumulating evidence has demonstrated that MitoQ plays a protective role in a variety of diseases [4]. For example, MitoQ has been reported to protect against renal ischemia-reperfusion (I/R) injury [5]. Neuzil et al. [6] have confirmed that MitoQ gives protection against I/R injury in the heart. It is reported that MitoQ could protect against organ damage in a rat model of sepsis [7]. However, the effect of MitoQ in sepsis-induced ALI has been poorly studied. Thus, more researches are still needed to further investigate the mechanisms of MitoQ on sepsis-induced ALI.

Previous studies have confirmed that, in the course of ALI, a large number of inflammatory factors such as interleukin-6 (IL-6), IL-1*β*, and tumor necrosis factor-*α* (TNF-*α*) are secreted by alveolar macrophages [8]. Recently, researches have shown that the phosphatidylinositol 3′-kinase/protein kinase B/mammalian target of rapamycin (PI3K/Akt/mTOR) pathway could regulate multiple physiological activities such as cell proliferation and apoptosis [9]. In addition, the PI3K/Akt signaling pathway has an important role in cell survival and oxidative stress in pulmonary inflammation [10]. Previous studies have suggested that hesperidin induces apoptosis and autophagy through inhibition of PI3K/Akt/mTOR and glycogen synthase kinase-3 beta (GSK-3*β*) pathways in colon cancer [11]. DEX has been reported to exert a protective effect on LPS-induced ALI rats via PI3K/Akt/mTOR pathways [10]. Xue et al. [12] have indicated that MitoQ10 could ameliorate pancreatic fibrosis through the mTOR pathway. However, the role of MitoQ in sepsis-induced ALI is still unknown.

In this research, we investigated the protective effect of MitoQ on sepsis-induced ALI and its related molecular mechanisms. Our results revealed that MitoQ could protect acute lung injury in septic rats by activating the PI3K/Akt/GSK-3*β*/mTOR pathway. Findings of our study may provide new theoretical foundation for deeply exploring the treatment of sepsis-induced ALI by using MitoQ.

## 2. Materials and Methods

### 2.1. Animals and Groups

Forty healthy male SD rats (320–370 g) were obtained from Nanjing Military Region General Hospital Animal Experimental Center. All animals were housed in 12 h light-dark cycle conditions with free access to food and water. All animal experiments in our study were approved by the ethics committee of our hospital. Animals were randomly divided into 4 groups (*n* = 10): control group (Con group), model group (CLP group), model + MitoQ group (MitoQ group), and model + MitoQ + PI3K inhibitor group (MitoQ + LY294002 group).

### 2.2. Establishment of Septic ALI Model and the Survival Rate of Septic ALI Rats

The septic ALI model was induced by cecal ligation and puncture (CLP). Briefly, animals were fasted for 12 hours before surgery. The rats in CLP group, MitoQ group, and MitoQ + LY294002 group were anesthetized with pentobarbital sodium (50 mg/kg). The abdomens of rats were shaved, and their cecums were exposed by a 2 cm abdominal midline incision. The cecum was isolated and ligated below the ileocecal valve, punctured by using a 21-gauge needle. Next, faeces were squeezed through the puncture wound. Finally, the cecum was returned to the peritoneal cavity and abdominal incision was then sutured. Rats in the Con group were only treated with laparotomy and distal cecum isolation. After operation for 0 h and 6 h, rats in the Con group and the CLP group were treated with normal saline (1 mL) by intraperitoneal injection, respectively. Meanwhile, rats in the MitoQ group were treated with MitoQ (2 mg/kg, Cell Signaling, USA) by intraperitoneal injection. In addition, rats in MitoQ + LY294002 group were intraperitoneally injected with MitoQ (2 mg/kg) and LY294002 (20 *μ*mol/L, Sigma, USA), respectively. After CLP treatment, the survival of the rats was recorded at 0, 3, 6, 12, and 24 h, and the survival rate was calculated.

### 2.3. The Wet/Dry (W/D) Weight Ratio of Rat Lung Tissue

Twenty-four hours after modeling, the right lower lobe of the rats in the Con group, the CLP group, and the MitoQ group was collected. Firstly, the liquid on the surface of the lungs was sucked up with absorbent paper, and the wet weight of the lung tissue was weighed. Then, the lung lobe was placed in an 80°C dryer for 72 h, and then the dry weight of the lung tissue was weighed. Finally, the ratio of W/D of the lung tissue was calculated.

### 2.4. Detection of High-Mobility Group Box 1 (HMGB1) and IL-6 Levels in Serum by ELISA

After modeling for 24 h, femoral vein blood (2 mL) was collected and centrifuged for 10 min. The levels of HMGB1 and IL-6 in serum were measured using an ELISA kit (Biosource International, Vancouver, USA), and all procedures were performed in strict accordance with the ELISA kit instructions.

### 2.5. Detection of Myeloperoxidase (MPO) Activity and Levels of MIP2 and KC in Lung Tissue

The homogenates of lung tissue were centrifuged to obtain supernatant for the following experimental detections. The activity of MPO was measured by the MPO colorimetric activity assay kit (Nanjing Jiancheng Co., Ltd., China) according to the manufacturer's instructions. Finally, the absorbance was measured at 460 nm to evaluate the MPO activity. The levels of MIP2 and KC in supernatant were measured using an ELISA kit (Biosource International), and all procedures were performed in strict accordance with the kit instructions.

### 2.6. HE Staining and Lung Injury Score

After fixed in 10% neutral formaldehyde solution for 24 h, the lung tissue was routinely dehydrated, transparent, and waxed. Next, paraffin-embedded lung tissue was cut into 4 *μ*m thick sections. The sections were dewaxed by xylene, dehydrated with ethanol of different gradient concentrations, and then stained with hematoxylin for 5 min and eosin stained for 2 min. Finally, a neutral resin was mounted and the histopathological changes were observed under an optical microscope. Tissue damage was scored according to a previous study [13].

### 2.7. Terminal Deoxynucleotidyl Transferase-Mediated dUTP Nick End Labelling (TUNEL) Staining

TUNEL staining assay was applied to detect the apoptotic cells in lung tissue samples. Briefly, lung tissues were fixed with 4% paraformaldehyde, embedded in paraffin wax, and sectioned into 4 *μ*m thick slices. The tissue section was then dewaxed and rehydrated. Subsequently, the apoptosis rate in lung tissue sections was detected by using the Tunel Kit (Shanghai Ruisai Biotechnology Co., Ltd., China). Finally, 5 random fields were selected in each section and the rate of positive cells was calculated under 400x magnification.

### 2.8. Western Blot Analysis

The lung tissues of each group were collected. Then, the protein sample was separated by 10% SDS-PAGE and transferred onto nitrocellulose membranes. The membranes were blocked with 5% skim milk for 2 h at room temperature and then incubated with primary antibody (p-Akt, Akt, p-mTOR, mTOR, Bax, Caspase-3, Caspase-9, LC-3, 1 : 1000, Cell Signaling, USA; p-GSK-3*β*, GSK-3*β*, 1 : 1000, Santa Cruz Animal Health, USA; Beclin-1, 1 : 1000, Thermo Fisher Scientific, USA) overnight at 4°C. Subsequently, the peroxidase-labeled secondary antibody (anti-rabbit IgG, 1 : 5000, Cell Signal, USA) was used for incubation for 2 h. The protein blots were visualized with an enhanced chemiluminescence (ECL) kit. Finally, the density of western blot bands was analyzed using Quantity One 1-D Analysis Software (Bio-Rad, USA).

### 2.9. Statistical Analysis

All statistical analyses were performed using SPSS 22.0 Statistical Software (Chicago, IL). The results were presented as the mean ± SD. The differences between various groups were analyzed by a one-way ANOVA followed by Tukey's post hoc test, and the data of the two groups were assessed using Student's *t* test. Survival rate data were analyzed by the Kaplan–Meier curve, and the log-rank statistical test was applied to compare the curves. All experiments were repeated three times. *P* < 0.05 was considered to be statistically significant.

## 3. Results

### 3.1. MitoQ Increases Survival Rate in Septic ALI Rats

**Figure 1:**
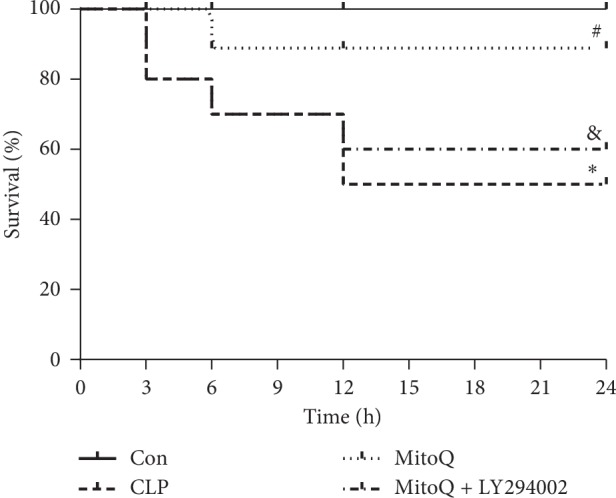
Effect of MitoQ on survival rate of sepsis-induced acute lung injury (ALI) in rats. The survival rates of rats were observed within 24 h. Results are expressed as percent survival, *n* = 10. The survival rate was analyzed by the Kaplan–Meier curve, and the log-rank statistical test was applied to compare the curves. ^*∗*^*P* < 0.05 versus Con group; ^#^*P* < 0.05 versus cecal ligation and puncture (CLP) group; ^&^*P* < 0.05 versus MitoQ group.

The survival rate of the rats in CLP group was markedly lower than that in the Con group (*P* < 0.05), while the survival rate in the MitoQ group was distinctly higher than that in the CLP group (*P* < 0.05). However, the intraperitoneal injection of LY294002 exacerbated the death of the ALI rats (Figure 1). These results suggested that MitoQ could improve the survival rate of septic ALI rats, while PI3K inhibitor LY294002 decreased the survival rate of septic ALI rats.

### 3.2. MitoQ Alleviates Pulmonary Edema and Inhibits Inflammatory Response

**Figure 2:**
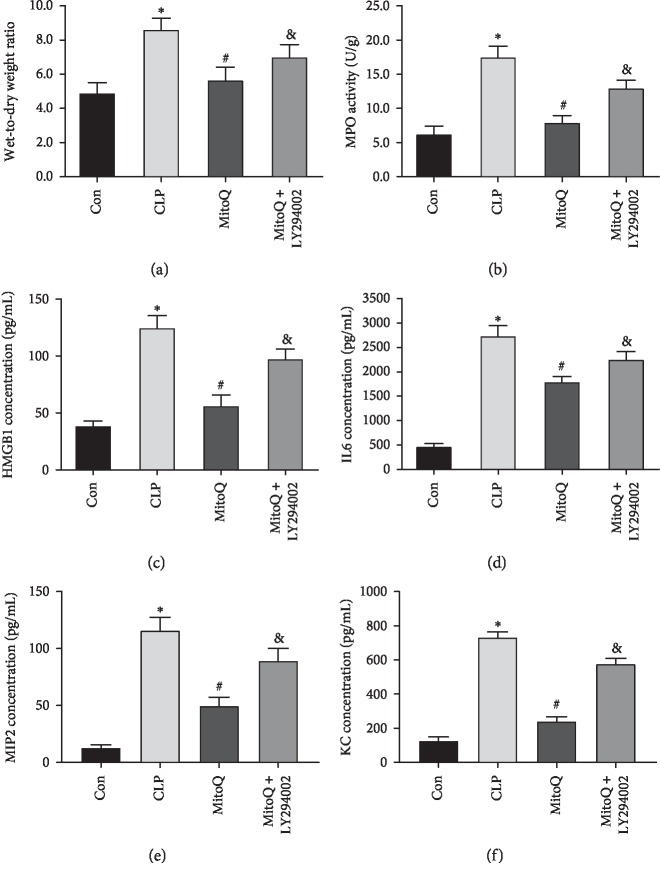
Effect of MitoQ on the pulmonary edema and levels of inflammatory factors in lung tissue of septic ALI rats. (a) The pulmonary edema was detected by the W/D weight method. (b) The activity of MPO was measured by the MPO colorimetric activity assay kit. (c) HMGB1 level was measured by ELISA. (d) IL-6 level was detected by ELISA. (e) MIP2 level was detected by ELISA. (f) KC level was detected by ELISA. Data are presented as mean ± standard deviation, repeated for three times. ^*∗*^*P* < 0.05 versus Con group; ^#^*P* < 0.05 versus CLP group; ^&^*P* < 0.05 versus MitoQ group.

When compared with Con group, the W/D ratio of lung tissue was significantly increased in the CLP group (*P* < 0.05). After MitoQ treatment, the W/D ratio of lung tissue was decreased markedly (*P* < 0.05). However, LY294002 significantly increased the W/D ratio of lung tissue (*P* < 0.05, Figure 2(a)). Those results illustrated that MitoQ could alleviate pulmonary edema in the sepsis-induced acute lung injury rats, while the PI3K inhibitor LY294002 aggravated pulmonary edema.

The activity of MPO and levels of HMGB1, IL-6, MIP2, and KC in CLP groups were prominently increased than those in the Con group, while the increase of MPO activity and the high levels of HMGB1, IL-6, MIP2, and KC were distinctly reduced by MitoQ treatment (*P* < 0.05). However, the effect of MitoQ was reversed by LY294002 (Figures 2(b)–2(d)). The results suggested that MitoQ could reduce the inflammatory response of lung tissue in the sepsis-induced acute lung injury rats and LY294002 increased the inflammatory response of lung tissue.

### 3.3. MitoQ Activates PI3K/Akt/GSK-3*β*/mTOR Signaling Pathway

**Figure 3:**
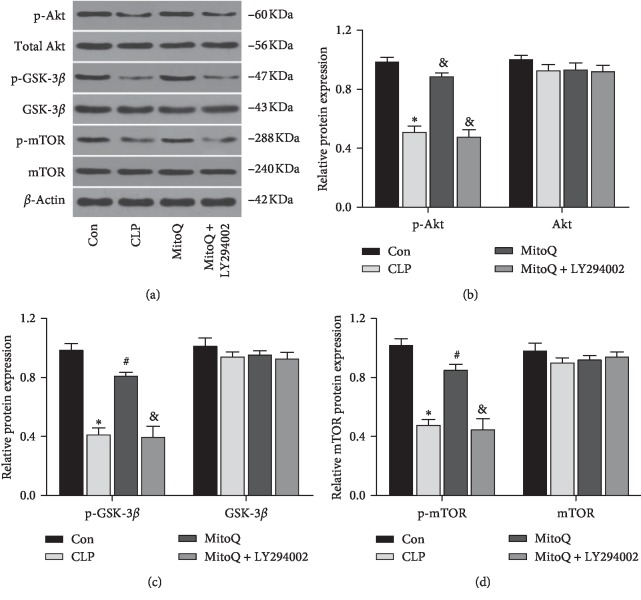
Effect of MitoQ on the PI3K/Akt/GSK-3*β*/mTOR pathway in lung tissue of septic ALI rats. (a) Expressions of p-Akt, Akt, p-GSK-3*β*, GSK-3*β*, p-mTOR, and mTOR were tested by western blot. Quantitative data of the levels of (b) p-Akt and Akt, (c) p-GSK-3*β* and GSK-3*β*, and (d) p-mTOR and mTOR. Data are presented as mean ± standard deviation, repeated for three times. ^*∗*^*P* < 0.05 versus Con group; ^#^*P* < 0.05 versus CLP group; ^&^*P* < 0.05 versus MitoQ group.

The results of the PI3K/Akt/GSK-3*β*/mTOR pathway-related protein expression and its phosphorylation level are shown in Figures 3(a)–3(d). The proteins expressions of p-Akt, p-GSK-3*β*, and p-mTOR in the CLP group and MitoQ + LY294002 group were significantly decreased compared with those of the Con group (*P* < 0.05). When compared with the CLP group, p-Akt, p-GSK-3*β*, and p-mTOR expressions were markedly increased in the MitoQ group (*P* < 0.05). Meanwhile, the expressions of p-Akt, p-GSK-3*β*, and p-mTOR were evidently decreased in the MitoQ + LY294002 group compared with the MitoQ group (*P* < 0.05). However, the levels of Akt, GSK-3*β*, and mTOR between different groups had no significant difference (*P* > 0.05).

### 3.4. MitoQ Inhibits Sepsis-Induced Acute Lung Injury in Rats

**Figure 4:**
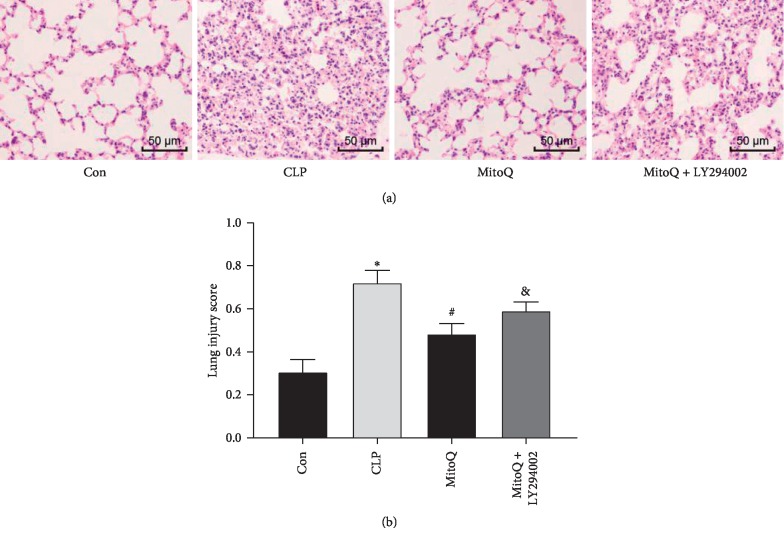
Effect of MitoQ on histopathological changes of lung tissue in septic ALI rats. (a) Histopathological changes of lung tissue in septic ALI rats with HE staining (magnification ×100). (b) Lung injury score of septic ALI rats. ^*∗*^*P* < 0.05 versus Con group; ^#^*P* < 0.05 versus CLP group; ^&^*P* < 0.05 versus MitoQ group.

Lung tissue sections were observed under a light microscope as shown in Figure 4(a). The alveolar structure of the Con group was clear and complete, and occasionally fewer inflammatory cells were observed in some areas. In the CLP group, the alveolar wall of most areas was broadened with edema and the alveolar cavity collapsed. Meanwhile, a large amount of inflammatory cell infiltration and erythrocyte exudation were also observed in the alveolar wall and the alveolar cavity in the CLP group. After treatment with MitoQ, the alveolar wall was slightly broadened. At the same time, inflammatory cell infiltration and red blood cell exudation were alleviated. In the MitoQ + LY294002 group, alveolar wall edema, inflammatory cell infiltration, and red blood cell exudation were more severe, which was aggravated compared with those of the MitoQ group but lessened than the CLP group. As shown in Figure 4(b), lung injury score in the CLP group were significantly increased compared with the Con group (*P* < 0.05). Compared with the CLP group, the lung injury score of the MitoQ group was significantly reduced (*P* < 0.05). Intraperitoneal injection of LY294002 significantly increased lung injury in the MitoQ + LY294002 group (*P* < 0.05).

### 3.5. MitoQ Inhibits Cell Apoptosis in Rat Lung Tissue

**Figure 5:**
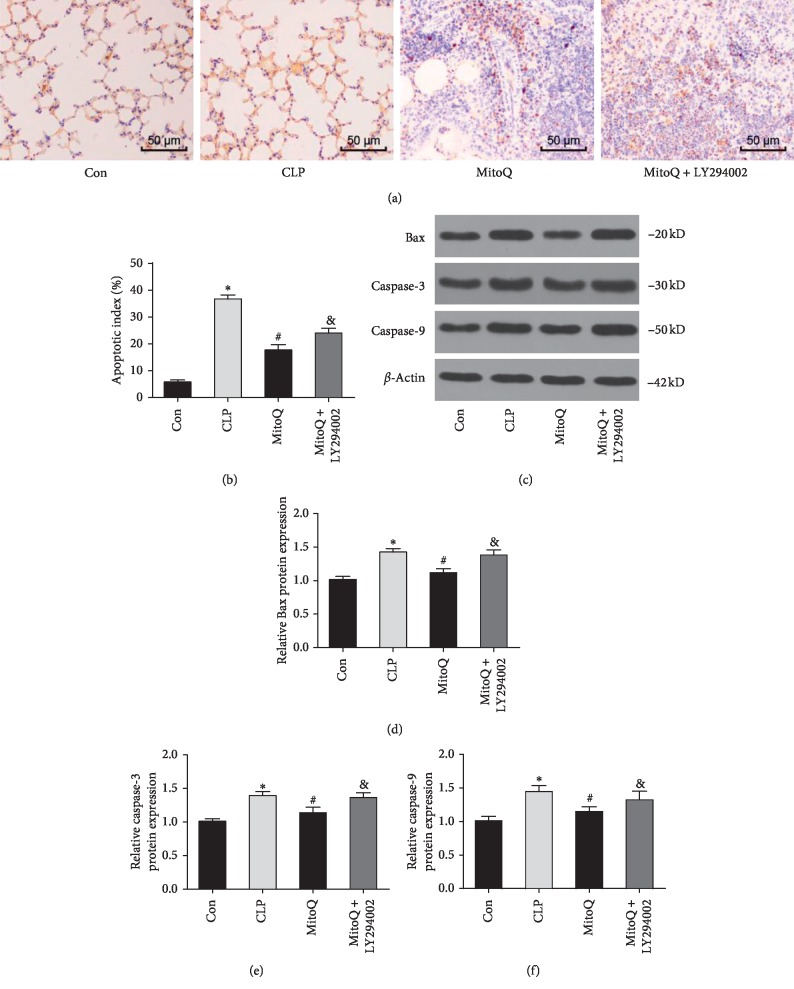
Effect of MitoQ on cell apoptosis in lung tissue of septic ALI rats. (a) Cell apoptosis was detected by TUNEL staining (×400). (b) Quantitative data of cell apoptosis index. (c) Expressions of Bax, Caspase-3, and Caspase-9 were tested by western blot. Data are presented as mean ± standard deviation, repeated for three times. ^*∗*^*P* < 0.05 versus Con group; ^#^*P* < 0.05 versus CLP group; ^&^*P* < 0.05 versus MitoQ group.

The apoptosis of lung tissue was detected by TUNEL staining, and the apoptotic cells were stained brown-yellow, and normal and necrotic lung tissue cells were not stained. Figures 5(a) and 5(b) show that the lung tissues of the Con group had only a small amount of positive staining and less apoptotic cells. Meanwhile, a large number of apoptotic cells were found in the CLP group, and the apoptotic index was significantly higher than that in the Con group (*P* < 0.05). Moreover, the apoptotic index of the MitoQ group was observably lower than that of the CLP group (*P* < 0.05). However, after LY294002 intervention, the effect of MitoQ was significantly reversed (*P* < 0.05).

When compared with the Con group, the expressions of Bax, caspase-3, and caspase-9 were evidently upregulated after CLP stimulation (*P* < 0.05). After treatment with MitoQ, the expressions of Bax, caspase-3, and caspase-9 protein were significantly decreased (*P* < 0.05). Compared with the MitoQ group and Con group, the expressions of apoptotic proteins in the MitoQ + LY294002 group were markedly increased (*P* < 0.05) (Figure 5(c)). These results indicated that MitoQ could inhibit the apoptosis of lung tissue cells in septic ALI rats by downregulating the expression of Bax, caspase-3, and caspase-9 protein.

### 3.6. MitoQ Inhibits Cell Autophagy in Rat Lung Tissue

**Figure 6:**
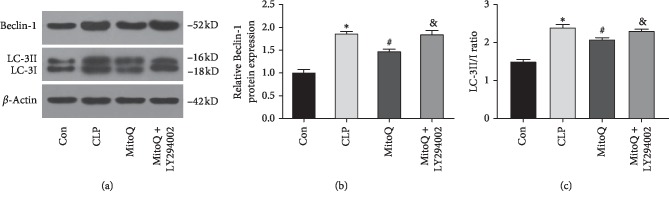
Effect of MitoQ on cell autophagy in lung tissue of septic ALI rats. (a) Expressions of Beclin-1 and LC-3 were tested by western blot. Quantitative data of the levels of (b) Beclin-1 and (c) LC-3II/LC-3I. Data are presented as mean ± standard deviation, repeated for three times. ^*∗*^*P* < 0.05 versus Con group; ^#^*P* < 0.05 versus CLP group; ^&^*P* < 0.05 versus MitoQ group.

Western blot was used to detect the effect of different treatments on the expression of autophagy protein Beclin-1 and the value of LC-3II/I in lung tissue (Figures 6(a)–6(c)). Compared with the Con group, the expression of Beclin-1 protein in the CLP group and the MitoQ + LY294002 group was markedly increased (*P* < 0.05, Figure 6(b)), and the values of LC-3II/I had the same trend (*P* < 0.05, Figure 6(c)). When compared with the CLP group, Beclin-1 expression and the values of LC-3II/I were markedly decreased in the MitoQ group (*P* < 0.05). Meanwhile, Beclin-1 expression and the values of LC-3II/I were evidently increased in MitoQ + LY294002 group compared with those in MitoQ group (*P* < 0.05). All the results indicated that MitoQ could inhibit the cell autophagy of lung tissue in septic ALI rats by downregulating Beclin-1 protein expression and LC-3II transformation.

## 4. Discussion

Sepsis, a complex systemic inflammatory response syndrome, is caused by an uncontrolled responses of the host to invading pathogens [14]. Sepsis-induced ALI or acute respiratory distress syndrome (ARDS) is the leading cause of mortality in septic patients [15]. Sepsis-induced ALI presents a series of clinical diseases, including pulmonary inflammatory responses and alveolar injury [16, 17]. Although many progresses have been made in the field of sepsis, the treatment effect of sepsis-induced ALI is limited.

Lung edema, inflammatory cells infiltration, and alveolar hemorrhage are considered as the typical pathological manifestations of ALI [18, 19]. MitoQ is beneficial to the treatment of sepsis. For example, previous studies have identified that MitoQ treatment could ameliorate organ damage in sepsis [7]. In our study, MitoQ increased survival rate and alleviated pulmonary edema and inflammatory cells infiltration in septic ALI rats, suggesting that MitoQ could attenuate ALI induced by sepsis.

Recently, ALI induced by sepsis has been reported to lead to lung cell apoptosis [16]. MitoQ exhibits antiapoptosis ability in a variety of diseases. For example, Zhou et al. [20] have revealed that the treatment with MitoQ significantly alleviated brain edema and inhibited cortical neuronal apoptosis. Similarly, Tunel assay results showed that MitoQ could inhibit septic ALI-induced cell apoptosis in our study. In addition, the expressions of apoptosis related proteins were also measured. The proapoptotic protein Bax, an important member of Bcl-2 protein family, plays a vital role in the caspases activation and mitochondrial apoptosis pathway [21]. In the present study, we confirmed that the high expressions of Bax, caspase-3, and caspase-9 induced by CLP were reversed by MitoQ treatment. Furthermore, previous researches have indicated that MitoQ could regulate autophagy by inducing a pseudomitochondrial membrane potential [22]. The expression of Beclin-1 and the transformation of LC3I to LC3II are two commonly used markers of cell autophagy. In this study, MitoQ downregulated Beclin-1 protein expression and the conversion of LC-3I to LC-3II, suggesting that MitoQ could inhibit the cell autophagy of lung tissue in septic ALI rats.

Lung parenchyma injury is characterized by the release of inflammatory cytokines, such as IL-6, IL-1*β*, and TNF-*α* [10, 23]. MitoQ has been reported to decrease the production of the proinflammatory cytokines, such as TNF-*α*, IL-1*β*, and IL-6 [24]. High-mobility group box 1 (HMGB1), a nuclear nonhistone DNA-binding protein, has an important role in inflammatory progression [25]. Meng et al. [10] have confirmed that the expressions of inflammatory cytokines is closely associated with HMGB1 regulation. In addition, the degree of activation of MPO has been considered as an indicator of the level of inflammation in lung tissue [26]. Our results showed that MitoQ could reduce MPO activity and the levels of HMGB1 and IL-6, which is consistent with the previous researches.

The PI3K/Akt/GSK-3*β*/mTOR signaling pathway is ubiquitous in mammal cells and involves an important various cellular activities, such as cell proliferation, apoptosis, and autophagy [9, 11, 27, 28]. The previous studies have suggested that hesperidin induces apoptosis and autophagy through inhibition of the PI3K/Akt/GSK-3*β*/mTOR pathway in colon cancer [11]. HMGB1 has been proven to regulate the PI3K/Akt pathway in myocardial ischaemia/reperfusion injury [29]. Xu et al. [30] have indicated that hydrogen sulfide ameliorates LPS-induced ALI through the PI3K/Akt/mTOR pathway in mice. The previous researches have reported that arctiin could protect against LPS-induced ALI via the inhibition of the PI3K/Akt pathway [31]. Moreover, MitoQ10 has been reported to attenuate aspects of diabetic cardiomyopathy by regulating PI3K signaling [32]. A study of Choi et al. [33] has indicated that MitoQ10 could protect against neuronal cell death by inhibiting the activation of the PI3K pathway. In our study, we investigated whether MitoQ could play a role in sepsis-induced ALI through the PI3K/Akt/GSK-3*β*/mTOR signaling pathway and found that the PI3K/Akt/GSK-3*β*/mTOR pathway could be activated by MitoQ treatment.

However, there are still some limitations in the current study. First of all, the detailed mechanism of MitoQ in sepsis was not fully investigated. Second, the survival experiment of MitoQ on septic rats was performed only within 24 h. Therefore, in order to determine the long-term and stable therapeutic benefits of MitoQ on sepsis, future studies on a longer time survival trial should be conducted.

## 5. Conclusions

MitoQ could protect sepsis-induced acute lung injury through the activation of the PI3K/Akt/GSK-3*β*/mTOR pathway in rats. These results may provide a theoretical basis for understanding the mechanism of MitoQ on sepsis-induced acute lung injury and further studies are still needed in the future.

## Data Availability

The data used to support the findings of this study are available from the corresponding author upon request.
